# Interlaboratory assessment of candidate reference materials for lentiviral vector copy number and integration site measurements

**DOI:** 10.1016/j.omtm.2025.101472

**Published:** 2025-04-21

**Authors:** Hua-Jun He, Zhiyong He, Steven P. Lund, Laure Turner, Yongjun Fan, Yu Qiu, David C. Corney, Boro Dropulic, Rimas Orentas, Oxana Slessareva, Priscilla Welch, Katie Dungca, Ellen Stelloo, Gabrielle Dijksteel, Harma Feitsma, Sana Ahmed-Seghir, Rostyslav Makarenko, Engin Altunlu, Daniëlle Steenmans, Jan Spanholtz, Monica Raimo, Shai Senderovich, Barbara S. Paugh, Chieh-Yuan Li, Benjamin Schroeder, Alexandra S. Whale, Dilek Yener, Carole A. Foy, Shareef Nahas, Feng Tu, Michael Sheldon, Yan Ding, Jennifer Kandell, Uma Lakshmipathy, Jennifer H. McDaniel, Justin M. Zook, Sierra Miller, Samantha Maragh, Simona Patange, Mahir Mohiuddin, Alessandro Tona, Kenneth D. Cole, Sheng Lin-Gibson

**Affiliations:** 1 National Institute of Standards and Technology, 100 Bureau Drive, Gaithersburg, MD 20899, USA; 2 GENEWIZ from Azenta Life Sciences, 111 Corporate Boulevard, South Plainfield, NJ 07080, USA; 3 Caring Cross, 910 Clopper Road, Gaithersburg, MD 20878, USA; 4 Castle Creek Biosci, 405 Eagleview Boulevard, Exton, PA 19341, USA; 5 Cergentis, a Solvias Company, Yalelaan 62, 3584 CM Utrecht, the Netherlands; 6 Genomic Vision, 80 Rue des Meuniers, Bagneux, Paris Area 92220, France; 7 Glycostem Therapeutics, Kloosterstraat 9, 5349 AB Oss, the Netherlands; 8 GSK, Gunnels Wood Road, Stevenage SG1 2NF, UK; 9 Lentigen, a Miltenyi Biotec Company, 1201 Clopper Road, Gaithersburg, MD 20878, USA; 10 Mission Bio, 400 E Jamie Court, South San Francisco, CA 94080, USA; 11 National Measurement Laboratory (NML at LGC), LGC, Queens Road, Teddington, Middlesex TW11 0LY, UK; 12 Sampled, 30 Knightsbridge Road, Piscataway, NJ 08854, USA; 13 Thermo Fisher Scientific, 10421 Wateridge Circle, San Diego, CA 92121, USA

**Keywords:** lentiviral vector, LV, vector copy number, VCN, integration site analysis, next generation sequencing, NGS, reference material, gene therapy, CAR T-cell therapy, digital PCR, interlaboratory testing, integration profiling

## Abstract

While lentiviral vectors have played a critical role in the emergence of gene-modified cell therapies, safety concerns remain regarding potential insertional mutagenesis. Regulatory authorities strongly recommend risk assessment and management of vector copy numbers (VCNs), integration profiles, and integration sites in the lentivirus-based cell and gene therapy products. However, accurately measuring these parameters remains a significant challenge due to the lack of standardized methodologies and VCN reference materials (RMs). Toward this challenge, we conducted an interlaboratory study on NIST candidate RMs for VCN measurements. The candidate RMs comprise five human genomic DNA samples or fixed cells from clonal Jurkat cell lines with defined VCNs ranging from 0 to 4. All 12 study participants were able to identify the VCN in the five blinded samples using quantitative PCR (qPCR), digital PCR (dPCR), or next generation sequencing (NGS) assays. Consensus value of VCN and integration sites in these candidate RMs were achieved. The fixed clonal VCN cells were also used to evaluate an emerging imaging-based technology called molecular combing. This interlaboratory assessment demonstrated the utility, commutability, and suitability of the NIST VCN candidate RMs for quality assurance and improved confidence in VCN, integration profile, and integration site measurements.

## Graphical abstract

**Figure:**
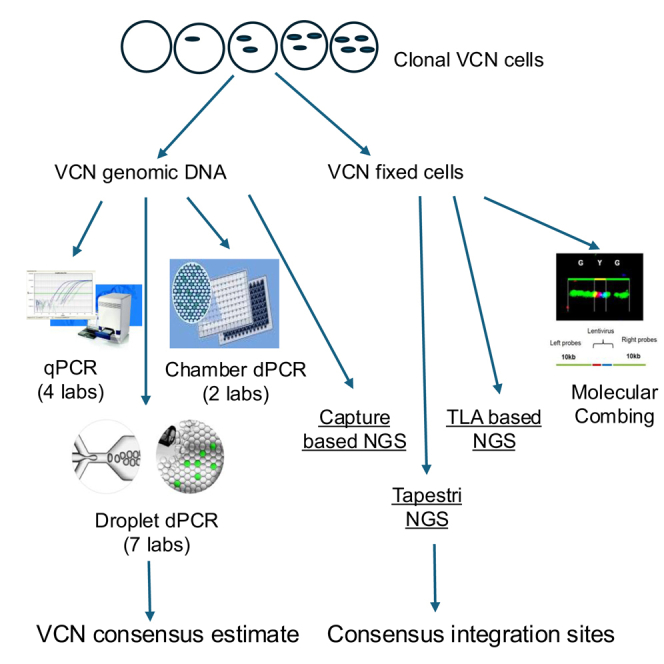

## Teaser

An NIST candidate lentiviral vector copy number (VCN) reference material was assessed by an interlaboratory study. The consensus VCN value could be used for assay harmonization in VCN measurements and consensus integration sites could be used to benchmark and improve the NGS technologies and bioinformatics pipelines.

## Introduction

Lentiviruses have the ability to transduce both dividing and nondividing cells^1^^,^^2^^,^^3^ and integrate their genetic load into the host genome, which makes lentiviral vectors (LVs) powerful tools for many applications.^4^^,^^5^ As of November 2024, there are 229 clinical trials worldwide using LVs, and 97 of them are CAR-T cell trials (https://ClinicalTrials.gov). One of the most important applications of LVs is gene and cell therapy.^6^^,^^7^^,^^8^^,^^9^^,^^10^^,^^11^^,^^12^^,^^13^ First, LVs can transduce a broad range of human cell types, including nonproliferating human monocytes.^14^ Second, LVs integrate their genetic load into the host genome and enable long-term expression of transgene in the target cells, which is critical for diseases requiring long-term treatment and persistent efficacy.^3^ Since the viral packaging genes are not integrated into the host genome, the relevant proteins that may cause immunogenicity will not be expressed, thereby reducing host adverse reactions. The flexibility of LVs has demonstrated its utilities within cell and gene therapy for the treatment of monogenic diseases and adoptive therapies such as chimeric antigen T cell (CAR-T) therapy.^15^

LVs hold great promise and even with multiple generations of development aimed at minimizing risks, safety concerns still exist. The major risks of LVs include potential for insertional gene inactivation, mutagenesis, and/or oncogenicity.^16^^,^^17^ When integrating into the host genome, LVs have integration site preferences and integration is not completely random, but uncontrolled.^18^ High integration number per cell may increase the chance of adverse effects such as insertional mutagenesis, oncogenicity, and/or chromosome instability.^19^^,^^20^^,^^21^^,^^22^ Regulatory authorities such as the US Food and Drug Administration (FDA) require the reporting of the average number of integrations per cell, VCN, on the Certificate of Analysis (https://www.fda.gov/media/156896/download) and long-term follow-up on LV gene therapy products. Although the VCN release criterion should be justified based on a risk assessment, a general rule of thumb is that the inserted VCN should be less than five copies/cell (presentation by Dr. Vatsan from the FDA at the ISBioTech conference on March 7, 2017). Recently, the FDA announced that it is investigating 22 cases of secondary T cell cancer after CAR-T cell therapy.^23^ In one case, an integration site was identified in the second intron of the *SSU72* gene, while the second allele of the *SSU72* gene was undisturbed. The mechanism underlying this secondary T cell lymphomas remains unclear.^24^ The measurements of lentiviral VCN and integration sites, along with CAR or other transgene expression, are critical information in determining any possible biological or causal relationship with LV products. However, obtaining these measurements can be difficult, as they are challenging and not yet standardized.^23^

Measuring the VCN in cell and gene therapy products is extremely important. Current measurements of the LV integration copy number, including quantitative PCR (qPCR), digital PCR (dPCR), and next generation sequencing (NGS), still have limitations and are often inconsistent due to the complexity of the assays from different laboratories and lack of standards. Additionally, NGS methods, such as targeted sequence capture sequencing technology,^25^ CRISPR-enhanced viral integration site sequencing,^26^ were used to determine LV integration sites. Due to the lack of standardized NGS methods and bioinformatics pipeline, assessment of integration data through NGS still has technical difficulties and challenges for the integration site analysis.^27^ Therefore, reference materials (RMs) for VCN measurements are critical for harmonizing results and increasing the confidence in these measurements. Furthermore, these RMs can also provide positive controls for developing and validating new assays.

There are ongoing efforts to develop lentiviral VCN RMs for gene therapy. The World Health Organization (WHO) Standardization Committee and the National Institute for Biological Standards and Controls (NIBSC) in the United Kingdom have developed a panel of lentiviral genomic DNA-based standards comprising a negative control without lentiviral integration and a range of samples containing a couple to approximately 10 copies of the lentiviral genome in HEK293T cell lines (https://www.who.int/publications/m/item/WHO-BS-2019.2373). When this material was used in the WHO-organized VCN collaborative study, around 30% of the integration sites were not able to reach a consensus from the participants using four different sequencing-based methods (https://www.who.int/publications/m/item/who-bs-2022.2433). Especially, there was some variability in the identification of integration sites in high VCN samples, which is a bit problematic. This highlights the challenges and the needs for well-characterized RMs for analyses of VCN and LV integration sites. Another study established a panel of three clonal HCT116 cell lines containing one, two, or three integrated proviruses^28^ and we have reported the development of clonal Jurkat cell lines for a panel of four standards with one, two, three, or four copies of lentiviral vector(s).^29^ These collaborative efforts highlight the progress made thus far.

In this study, we developed a set of NIST candidate RMs that were human genomic DNA samples and fixed cells with defined VCN based on our panel.^29^ This study validates the utility and suitability of these materials for a variety of measurement methods in an interlaboratory study.

## Results

### Production summary of candidate VCN RMs

The materials for this interlaboratory study were prepared in two formats: a set of human genomic DNA samples and a set of fixed clonal cell lines with defined VCN (0, 1, 2, 3, and 4) per cell (hereafter named samples VCN0 to VCN4). To assess the individual laboratory performance, the samples were blinded and randomly labeled as samples A to E (sample A is VCN4, sample B is VCN2, sample C is VCN0, sample D is VCN3, and sample E is VCN1). Each DNA set consists of five vials containing genomic DNA extracted from the clonal VCN cell lines. The DNA was suspended in 1X TE buffer (10 mM Tris, 1 mM EDTA, pH 8.0) at a concentration of approximately 25 ng/μL (concentration was measured by absorbance described in materials and methods) and stored at 4°C. Each vial contains 80 μL of genomic DNA, resulting in total of approximately 2 μg DNA. Samples were stored at 4°C and shipped with cold packs. Each fixed cell set was prepared according to the protocols provided by participating laboratories, stored at 4°C, and shipped with cold packs.

### G-banded karyotyping

Results for all five clonal cell lines are in Table S2. The Jurkat parental clone (ATCC 6E−1) was a pseudodiploid cell line. The karyotype is 46,XY,-2,-18,del(2) (p21p23),del(18) (p11.2). Most cells had normal X and Y chromosomes. It shows that the genome is stable after the LV integration and all have the same karyotype except the VCN2 clone, which lost chromosome Y.

### VCN determination and stability evaluation

The VCN was calculated as the ratio of RRE (target sequence in proviral DNA) and *RPL32* (human reference gene, single copy in the haploid human genome) multiplied by two.^29^ The stability was evaluated by VCN measurements in randomly selected vials using Bio-Rad droplet dPCR (ddPCR) assays at different time points. The data did not show any substantial shift in VCN values (within the uncertainty of the measurements) for the five components for periods up to 810 days (Figure S1 in the supplemental material). The samples were stored at 4°C (range 4°C–6°C) in the dark for the indicated times before analysis. These data indicated that the DNA candidate RMs were stable for at least the tested period.

### Homogeneity assessment

**Figure 1:**
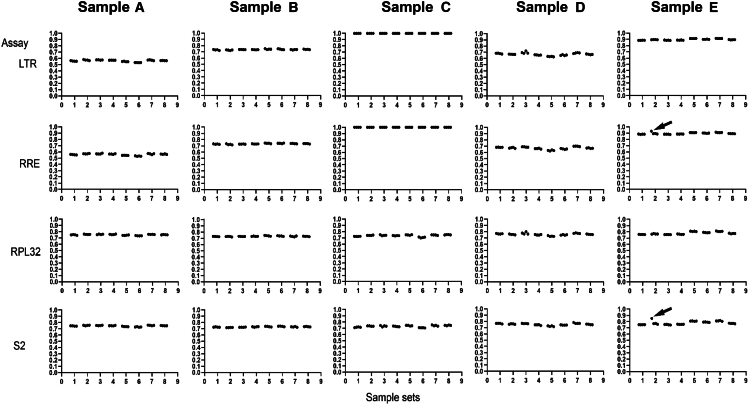
Homogeneity of VCN candidate RMs Homogeneity results of the eight sets of VCN candidate RMs. PCR-positive and PCR-negative droplets are counted to provide quantification of target DNA. Points in the plot are the proportion of negative droplets. Values on the plots show the proportion of negative droplets. The arrows indicate the data as known outliers recorded in the notebook due to mis-dispersing samples, and the corresponding observations were removed prior to analysis. Randomly selected sample set: (1) Set 2. (2) Set 9. (3) Set 33. (4) Set 55. (5) Set 67. (6) Set 78. (7) Set 88. (8) Set 102.

Each component of VCN candidate RMs was aliquoted into vials (160 vials for each component), then stored at 4°C (range 4°C–6°C) in the dark. Homogeneity studies were conducted by randomly selecting eight sample sets (sets #2, 9, 33, 55, 67, 78, 88, and 102) of each component, distributed throughout the order of dispensing. These samples were analyzed using the duplex Bio-Rad ddPCR assays to determine the copy number of human reference genes (*RPL32* and S2) and the target genes (LTR and RRE).^29^ The VCN was calculated as the ratio of target gene and reference gene multiplied by 2. The data did not indicate any obvious trend in the proportion of negative to positive values in any of the five components that varied with their dispensing order (Figure 1). The standard deviation corresponding to random set effects (i.e., set-to-set variability) was less than 1% of the estimated VCN for samples A, B, and D, and less than 2% of the estimated VCN for sample D, based on DerSimonian-Laird analyses (Table S1). VCN estimates for sample C were all 0 (i.e., no set-to-set or measurement variability was observed). The homogeneity of VCN values of all sample sets analyzed is shown in Figure S2 in the supplemental material. These findings confirm the consistent homogeneity of the VCN candidate RMs.

### Interlaboratory study

A total of 12 laboratories participated in this interlaboratory study. The participants were recruited through an online invitation for participation on the NIST website (https://www.nist.gov/programs-projects/integrated-lentiviral-vector-copy-number-measurements-invitation-participate) and through the NIST Flow Cytometry Standards Consortium and Genome Editing Consortium. Three principal methods, qPCR, dPCR (Table 1), and NGS (Table S3), were used by 11 laboratories for the quantification of LV integration copy numbers. One laboratory used an imaging-based method to evaluate the LV integration sites. Several laboratories submitted results using more than one method. Specifically, four laboratories used qPCR and seven laboratories used ddPCR (Bio-Rad QX series) with two also supplying data from chip-based dPCR (Qiagen QIAcuity), to evaluate lentiviral VCN. Three laboratories performed sequencing-based methods to determine both copy number and integration sites. All studies were performed using participants’ individual laboratories’ reagents and equipment. In terms of study protocols, all PCR data were obtained from participating laboratories’ (in-house) protocols or our published protocol.^29^ Some laboratories provided two sets of data for one method using different protocols. For every sample, each set of data comprised results from experiments tested at least in triplicate. The technologies and assay methods used by the participants are summarized in Tables 1 and S3.

**Table 1:** PCR-based technologies and methods used by interlaboratory study participants

Lab	Technology platform	Calibration control and instruments	Partition volume V_d_ (μL)	VCN calculation	Primer and probe sequence information
1	qPCR, standard curve	Bio-Rad CFX Opus 96, one linearized plasmid with vector and reference gene, duplex reactions		GAG/PTBP2 X2	GAG-Fwd: CATATAGTA TGGGCAAGCAGG GAG -Rev: CCCAGTAT TTGTCTACAGCCTTC GAG-Probe: FAM-ACG ATTCGCAGTTAATC CTGGCCT-BHQ 1 PTBP2-Fwd: TCTCCA TTCCCTATGTTCATGC PTBP2-Rev: GTTCCC GCAGAATGGTGAGGTG PTBP2-Probe: HEX-ATGTTCCTCGGAC CAACTTG-BHQ 1
2	qPCR, standard curve	Applied Biosystems QuantStudio 5, Psi-GAPDH gBlock, duplex reactions		HIV-Psi/GAPDH X 2	HIV-Psi-Fwd: ACTTGAA AGCGAAAGGGAAAC HIV-Psi -Rev: CACCCAT CTCTCTCCTTCTAGCC HIV-Psi-Probe: HEX-AGCTCTCTC/ZEN/GACGCAGGACTC GGC-IABkFQ GAPDH-Fwd: GTTGCCATCAAT GACCCCTT GAPDH-Rev: AAACCTGGGGG AATACGTGA GAPDH-Probe: FAM-TGTGGGAGG/ ZEN/AGCCACCTGGCTG -IABkFQ
3	qPCR, standard curve	Bio-Rad CFX Connect, vector plasmid + genomic DNA, SYBR Green-based reactions		RRE/ALB X 2	RRE-Fwd: AACTCACAG TCTGGGGCATC RRE-Rev: CAGCAGTG GTGCAAATGAGT ALB-Fwd: GAGCTTAT GGAGGGGTGTTTCA ALB-Rev: TCTGTTTG GCAGACGAAGCC
4A	qPCR, standard curve	Applied Biosystems ViiA 7, one linearized plasmid with vector and reference gene, duplex reactions		RRE/RPL32 X 2	See Paugh et al.^29^
4B	dPCR, Bio-Rad, Df x λ/Vd	Bio-Rad QX200 with Supermix (no dUTP) and duplex reactions	0.0007691	RRE/RPL32 X 2	See Paugh et al.^29^
4C	dPCR, QIAcuity, Df x λ/Vd	QIAGEN QIAcuity with duplex reactions	0.00034	RRE/RPL32 X 2	See Paugh et al.^29^
5A	dPCR, Bio-Rad, Df x λ/Vd	Bio-Rad QX200 with Supermix (no dUTP) and duplex reactions	0.000749	RRE/ALB X 2; RRE/RPL32 X 2	NIST-RRE and NIST -RPL32 see Paugh et al.,^29^ and LentiV-RRE see Zhao et al.^27^ ALB-Fwd: GCTGTCA TCTCTTGTGGGCTGT ALB-Rev: ACTCATGG GAGCTGCTGGTTC ALB-Probe: FAM-CCTGT CATGCCCACACAAAT CTCTCC-BHQ1
5B	dPCR, QIAcuity, Df x λ/Vd	QIAGEN QIAcuity with duplex reactions	0.00034	RRE/ALB X 2; RRE/RPL32 X 2	NIST-RRE and NIST -RPL32 see Paugh et al.,^29^ and LentiV-RRE see Zhao et al.^27^ ALB-Fwd: GCTGTCAT CTCTTGTGGGCTGT ALB-Rev: ACTCATG GGAGCTGCTGGTTC ALB-Probe: FAM-CCTG TCATGCCCACACAAA TCTCTCC-BHQ1
6	dPCR, Bio-Rad, Df x c/μL in rxn from software	Bio-Rad QX200 with Supermix (no dUTP) and duplex reactions	Software defined	HIV/RPLP0 X 2	Confidential, not available
7	dPCR, Bio-Rad, Df x λ/Vd	Bio-Rad QX200 with Supermix (no dUTP) and duplex reactions	Software defined	RRE/RPP30 X 2	NIST-RRE see Paugh et al.^29^; RPP30, HEX assay from Bio-Rad, Assay ID# dHsaCP2500350
8	dPCR, Bio-Rad, Df x c/μL in rxn from software	Bio-Rad QX ONE, duplex reactions	Software defined	RRE/GAPDH X 2	TaqMan Gene Expression Assays the lenti-specific target (RRE) and the housekeeping target (GAPDH) from Thermo Fisher
9	dPCR, Bio-Rad, Df x c/μL in rxn from software	Bio-Rad QX200 with Supermix (no dUTP) and duplex reactions	Software defined	RRE/RPL32 X 2; Integration site region/RPL32 X 2	See Paugh et al.^29^
10	dPCR, Bio-Rad, Df x c/μL in rxn from software	Bio-Rad QX200 with Supermix (no dUTP) and duplex reactions	Software defined	RRE/RPL32 X 2	See Paugh et al.^29^

Candidate RMs tested by participants with several qPCR, and dPCR assays are reported in this table. The VCN is calculated as 2-fold ratio of target in provirus vector to reference gene in the human genome.

### VCN measurement by qPCR

**Figure 2:**
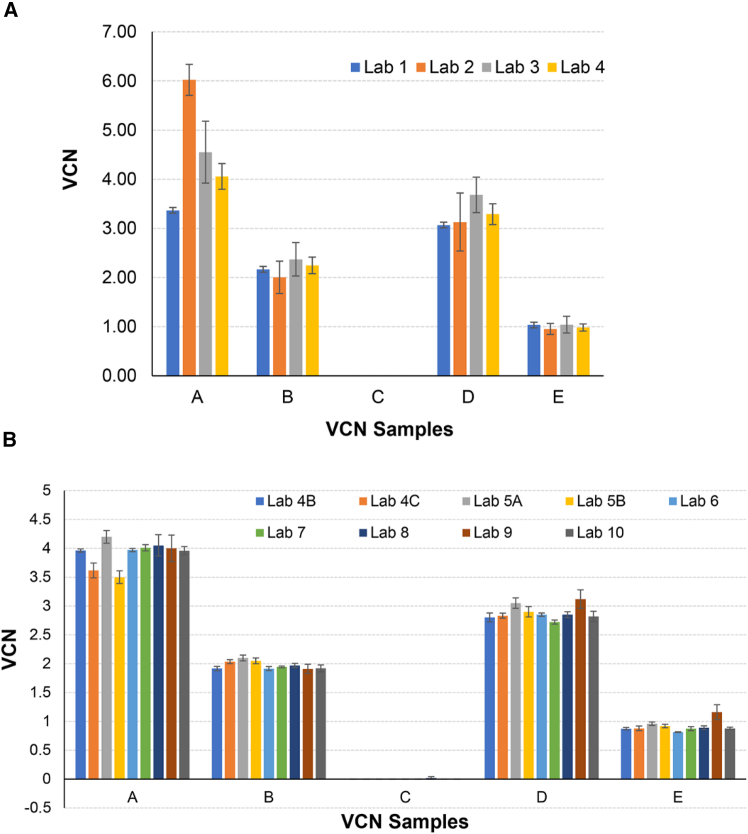
qPCR and dPCR interlaboratory study results (A) For qPCR, the copy concentration of target and reference genes were analyzed by using individual laboratories’ standard curve and raw Cq data. The VCN values were calculated based on the ratio of copy concentration of target and reference genes. The error bar is the standard deviation (*N ≥* 6). (B) For dPCR estimates calculated using individual laboratories’ protocol, such as provirus targets and reference gene selection. The error bar is the standard deviation (*N ≥* 3).

DNA quantification by qPCR requires a known copy number DNA calibrator to construct a standard curve. The DNA calibrators for standard curve constructions were provided by individual participants using synthetic plasmid DNA and/or genomic DNA. The quantification of VCN needs both the LV target gene and reference gene concentration, which were calculated from raw Cq (quantification cycle) value and standard curve. The qPCR amplification efficiency, an important indicator of the performance of a qPCR assay, was also calculated based on individual laboratories’ standard curves using the equation (qPCR efficiency $E=10^{\left(-\frac{1}{slope}\right)}-1$). The majority (three out of four) of qPCR assays gave a qPCR efficiency (E) within the acceptable range of 0.9–1.10. The large variation in the qPCR assays is likely to be the use of different plasmid DNAs and a combination of genomic DNA as a calibrator or standard to construct a standard curve for the qPCR analysis. Additionally, challenges in selecting suitable reference gene(s) may lead to the variation. The qPCR results are shown in Figure 2A. Overall, the qPCR results show no substantial differences between laboratories among the samples B–E, except sample A, or VCN4, which lab 2 overestimated by nearly 50%.

### VCN measurement by dPCR

dPCR has the capability to measure the absolute concentration of a target nucleic acid sequence in a background of non-target sequences, thus eliminating the need for calibration or use of standards. National Metrology Institutes are increasingly using dPCR to characterize and certify their RMs.^30^ In this study, two laboratories used QIAGEN QIAcuity dPCR (chamber/well-based dPCR) and seven laboratories used Bio-Rad ddPCR instrument (droplet-based dPCR) to measure lentiviral VCN. Figure 2B shows the dPCR results that the detected VCNs are very close to the expected numbers for all VCN 1–4 samples. Interlaboratory VCN variations are smaller for dPCR than that of qPCR. This phenome was also reported in the WHO-organized VCN collaborative study, (https://www.who.int/publications/m/item/who-bs-2022.2433). The difference in variations might be due to inherent variations in PCR chemistry and technology such as dPCR tends to be more resistant to PCR inhibitors relative to qPCR. It was also noted that the detected VCN value for sample A by QIAcuity was ∼8%–12% lower than the values using the Bio-Rad instruments. No bias was observed between instruments for the other four samples.

### Interlaboratory study consensus VCN values

The NIST Consensus Builder (https://consensus.nist.gov/) was used to estimate the consensus value of NIST candidate RMs from dPCR results. The DerSimonian-Laird procedure, a random-effects method, was employed to calculate the consensus values. Tables 2 and 3 show VCN and LV copy concentration consensus estimates with standard uncertainties, 95% CI, and dark uncertainty. For dPCR, partition size or volume is critical for the calculation of the copy concentration. However, it is not a trivial task to determine the partition size in either droplet or chip-based instruments, so it was understandable that many laboratories used the instrument default volume 0.85 nL for the Bio-Rad and 0.34 nL for the QIAcuity copy number calculations. For comparison purposes, all data were converted to the default volume by using a conversion factor value on the submitted copy number. These data could be readily converted when a more accurate copy concentration measurement is needed. The VCN is not impacted by partition volume as the metric is a ratio of two copy number concentrations.

**Table 2:** VCN consensus estimate by NIST Consensus Builder

	Sample A	Sample B	Sample C	Sample D	Sample E
Consensus estimate	4.02	1.96	0	2.85	0.882
Standard uncertainty	0.0342	0.0303	0	0.0457	0.0293
95% CI	3.96–4.09	1.9–2.02	0	2.76–2.94	0.825–0.94
Dark uncertainty	0.0773	0.072	0	0.108	0.071

NIST Consensus Builder: Fit laboratory effects model using DerSimonian-Laird procedure. The data from all seven ddPCR results using the Bio-Rad platforms were used to build the consensus.

**Table 3:** LV copy concentration consensus estimate by NIST Consensus Builder

	Sample A	Sample B	Sample C	Sample D	Sample E
Consensus estimate^a^	15,300	8,550	0	12,000	3,690
Standard uncertainty	1,010	318	0	668	155
95% CI	13,300–17,200	7,930–9,180	0	10,700–13,300	3,390–4,000
Dark uncertainty	2,410	745	0	1,590	347

NIST Consensus Builder: Fit laboratory effects model using DerSimonian-Laird procedure. The data from all seven ddPCR results using the Bio-Rad platforms were used to build the consensus.

a All ddPCR droplet sizes were recalculated to the same partition volume 0.85 nL for comparison.

### LV integration site analysis

Three laboratories evaluated LV integration sites for candidate materials using NGS-based methods following individual laboratories’ in-house protocols (Table S3). Table 4 shows that after resolving initial discrepancies, identical integration sites were detected by all three laboratories for 10 integration sites in five samples from VCN0 to VCN4. Surprisingly, the initial results from all three laboratories only reported partially identical integration sites. Strikingly, all three labs reported a site in chr16: 2,259,702 instead of the expected chr4: 5,612,738 for sample A (or VCN4), that was published previously when these clonal cell lines were established.^29^ After these NGS data were reanalyzed in each laboratory by their in-house optimized bioinformatics pipeline, they reported only a couple of discrepancies ranging from one base pair (bp) to several bps. To resolve these differences, we curated the data from each laboratory alongside whole genome sequencing data at each integration site using the genome browser IGV. In most cases, the differences were caused by differences in how sites were reported due to the duplication of 5 bp at the integration site. To further confirm the integration site on chromosome 16, we conducted two independent whole genome sequencing at 35–50X depth by short-read Illumina sequencing on sample A. Because the integration site is in an *SINE* near an LTR, which is similar to the previously reported chr4 region, the chr4 region may have been incorrectly reported due to mismapping of reads. Additionally, PacBio long-read sequencing was used to confirm these consensus results for sample A. In addition to the chr16 site, there are five other integration sites that are 1 bp to 80 bp different from the previously reported integration sites, which highlights the utility of these RMs for NGS assay development, optimization, and validation.

**Table 4:** Interlaboratory study provirus integration sites after curating data from each technology

Samples	*Sci Rep* paper^29^ (hg38)	Lab 9 (hg38)	Lab 10 (hg38)	Lab 11 (hg38)
A	chr1:45,634,499	chr1:45,634,495(+) - 45,634,499(−)	chr1:45,634,499-45,634,495	chr1:45,634,495(+)
	*chr1:160,440,748*^a^	chr1:160,440,776(+) - 160,440,780(−)	chr1:160,440,776-160,440,780	chr1:160,440,780(−)
	*chr4: 5,612,738*^a^	chr16:2,259,698(+) - 2,259,702(−)	chr16:2,259,702-2,259,698	chr16:2,259,702(−)
	chr22:42,082,676	chr22:42,082,672(+) - 42,082,676(−)	chr22:42,082,676-42,082,672	chr22:42,082,672(+)
B	*chr6:45,210,754*^a^	chr6:45,210,831(+) - 45,210,835(−)	chr6:45,210,831- 45,210,835	chr6:45,210,835(−)
	*chr22:40,844,112*^a^	chr22:40,844,212(+) - 40,844,216(−)	Chr22: 40,844,216-40,844,212	chr22:40,844,212(+)
C	not detected	not detected	not detected	not detected
D	*chr3:130,934,986*^a^	chr3:130,934,987(+) - 130,934,991(−)	chr3:130,934,991-130,934,987	chr3:130,934,987(+)
	chr14:22,564,447	chr14:22,564,447(+) - 22,564,451(−)	chr14:22,564,447-22,564,451	chr14:22,564,451(−)
	chr15:25,124,969	chr15:25,124,969(+) - 25,124,973(−)	chr15:25,124,969-25,124,973	chr15:25,124,973(−)
E	*chr3:35,632,784*^a^	chr3:35,632,785(+) - 35,632,789(−)	chr3:35,632,785-35,632,789	chr3:35,632,789(−)

a All of the italicized and underlined coordinators/integration sites reported in the Sci Rep paper differ from the integration sites identified in this interlaboratory study.

Lentiviral integration is mediated by the viral pre-integration complex, in which the reverse-transcribed viral genome is associated with the host chromatin and integrated into the host genome via the viral integrase.^31^ Two monomers of an HIV-1 integrase tetramer within the cleaved intasome use the viral DNA 3′-hydroxyl groups to cut the target DNA with a 5-bp stagger, which concomitantly joins the LTR ends to target DNA 5′ phosphates. Repair of the strand transfer complex yields a 5 bp duplication of target DNA flanking the integrated HIV-1 provirus.^31^ Table 5 showed the 5-bp duplications in the integration sites.

**Table 5:** Consensus provirus integration sites

Samples	Consensus integration sites (hg38)	Duplication sequence	Gene	Region
A	chr1:45,634,495(+) - 45,634,499(−)	GTTTG	GPBP1L1	Intron
	chr1:160,440,776(+) - 160,440,780(−)	TGTAC	non-coding	intergenic
	chr16:2,259,698(+) - 2,259,702(−)	TTAGC	RNPS1	Intron
	chr22:42,082,672(+) - 42,082,676(−)	GTTTT	SMDT1	Intron
B	chr6:45,210,831(+) - 45,210,835(−)	CAGAC	SUPT3H	Intron
	chr22:40,844,212(+) - 40,844,216(−)	GATAC	ST13	Intron
C	not detected	N/A	N/A	N/A
D	chr3:130,934,987(+) - 130,934,991(−)	CACCC	ATP2C1	Intron
	chr14:22,564,447(+) - 22,564,451(−)	CAAAT	non-coding	intergenic
	chr15:25,124,969(+) - 25,124,973(−)	CATAT	SNHG14	3′UTR
E	chr3:35,632,785(+) - 35,632,789(−)	ATAAC	non-coding	intergenic

N/A = not applicable.

### Integration sites determined by direct visualization

Molecular combing is an imaged-based method to untangle single DNA molecules, with a unique detection strategy, the Genomic Morse Code (GMC). This approach allows the direct visualization of large regions of interest (3 kbp–several Mbp). This technology was used to identify the integrated provirus at specific integration sites to discriminate the VCN cell lines by participating laboratory #12. The results are shown in Figure S3 in the supplemental material. This technology can detect the capture ratio of 1:1 of integration. No concatemer was detected; flanking regions were essential to distinguish between background and lentivirus integration due to the size of the lentivirus. Additionally, we could successfully confirm the heterozygosity of integration. These data verified the utility of these VCN candidate RMs to evaluate the performance of new technologies.

## Discussion

VCN analysis of transduced cells is a critical parameter for evaluating the efficacy and safety of LV-based gene therapy. Although qPCR is one of the principal methods for VCN measurement, there are reports indicating that qPCR assays can underestimate VCN values.^27^ To enhance the accuracy and precision of qPCR assays for VCN measurements, it is essential to use commutable standards and validated qPCR assays. Conversely, dPCR technique provides an endpoint absolute quantification, without the need for standards, and thus reducing susceptibility to variation. While dPCR is gaining widespread adoption, it requires optimization and has certain limitations and considerations such as uncertainty of partition volume and limited dynamic range for detection that users should be aware of when measuring VCN by dPCR.^30^ The candidate VCN RM is based on the backbone of third-generation lentiviral vectors, which could be useful for lentiviral vector designs carrying qPCR or dPCR assays targeting sequences. Furthermore, all clonal VCN cell lines share the same karyotype, except for the VCN2 clone, which has lost chromosome Y. The karyotype is 46,XY,-2,-18,del(2) (p21p23),del(18) (p11.2). Therefore, reference genes located on chromosomes other than chr2, chr18, and chrY might be suitable for VCN analysis as part of a "normal" diploid genome.

Since the advent of self-inactivating (SIN) vectors, clinical trial reports of insertional mutagenesis caused by LV integration have become rare. Nonetheless, it remains crucial to study vector integration sites and vector copy number to assess safety, prolonged genomic toxicity, and post-transcriptional deregulation events. The FDA is currently investigating 22 cases of secondary T cell cancer after CAR-T cell therapy. In one case, an integration site was identified in the second intron of the *SSU72* gene, while the second allele of the *SSU72* gene was undisturbed. The mechanism underlying this secondary T cell lymphoma remains unclear.^24^ Hematologic cancer developed in approximately 10% (7 of 67) of patients with cerebral adrenoleukodystrophy who received autologous hematopoietic stem-cell gene therapy with a lentiviral vector, predominant clones contained lentiviral vector insertions at multiple loci, including at either MECOM–EVI1 or PRDM16. These cases might associate with clonal vector insertions within oncogenes.^32^ These reports highlighted the unmet needs and challenges of measurements for gene and cell therapy products. Around 30% of the integration sites were not able to reach a consensus from the participants using four different sequencing-based methods in the WHO VCN collaborative study (https://www.who.int/publications/m/item/who-bs-2022.2433). In this study, one site was even misidentified on a different chromosome, and the other five sites were off by one to ∼80 bp in a total out of 10 sites.^29^ It is emphasizing ongoing challenges and the urgent need for standardized, effective methods and technologies for integration site detection. These well-characterized candidate RMs could be used to benchmark and improve the NGS technologies and bioinformatics pipelines for integration site analysis.

LV integration is uncontrolled but not random. Genome-wide studies of viral integration have shown that lentiviruses often integrate into actively transcribed genes, and that this preference is conserved across different species. Lens epithelium-derived growth factor (LEDGF)/p75, a lentiviral tethering protein, recruits the pre-integration complex to transcriptional units and facilitates integration. LEDGF/p75 binding sites are enriched in gene bodies and are mostly absent in promoters and intergenic regions, mirroring patterns of lentiviral integration.^33^ For the 10 integration sites we investigated, six were located in the introns, three were in the intergenic regions, and one in the 3′UTR. These results are consistent with previous observations that lentiviruses may prefer to integrate within intragenic regions, especially within introns.^34^ High-quality data from integration sites analyses may provide insight into how the integrase complex recognizes integration sites. Leveraging AI and high-quality data, there is potential to predict, modify, and target LV insertion toward safer genomic loci suitable for cell and gene therapy.

A new technology, molecular combing, has identified and confirmed LV at several specific integration sites in these defined VCN clonal Jurkat cell lines, demonstrating one of the utilities of these materials as references. The molecular combing technique, an image-based method, could be used to confirm or identify integrated proviruses at specific integration sites at the single-cell level. It can also be used to distinguish between tandem insertions and heterozygous or homozygous integrations. However, it requires prior knowledge of the integration sites, and it is a time-consuming and low-throughput technique. With the characteristics of defined integration sites, these materials may also be useful for development and validation of single-cell-based assays.^35^

The developed NIST VCN candidate RMs, and the interlaboratory results demonstrated their utility for harmonizing the VCN measurements for qPCR, dPCR, and NGS assays. Cell therapies are typically not clonal, and different cell populations might have different integration sites, which makes characterization by methods like NGS more challenging. Well-characterized clonal cell line materials can be used to evaluate emerging technologies and improve the confidence and accuracy of integration site analysis methods. However, even when using optimized NGS methods validated with these clonal VCN candidate RMs, we should exercise caution when identifying integration sites in complex samples.

The current candidate VCN RMs were derived from the ATCC Jurkat cell line and may be limited to research use only due to the ATCC material transfer agreement. In the future, we plan to collaborate with the gene and cell therapy community to develop NIST-immortalized human cell lines with minimal genome disruption from broadly consented donors. We also aim to develop and freely distribute reference materials derived from clonal CAR-T cell lines or other materials that address the community’s needs for VCN, integration sites, and CAR function studies.

## Materials and methods

### Ethics statement

The Jurkat cell line was derived from tissues from a 14-year-old patient with acute T cell leukemia in 1970s. The cell line was established prior to the current research use consent process. In 2013, The Cancer Genome Atlas (TCGA) steering committee administered by the National Cancer Institute (NCI) and National Human Genome Research Institute (NHGRI) at the National Institutes of Health (NIH) decided that the benefits outweighed the risks for making genomic data fully public without controlled access for Jurkat and other widely used, commercially available cell lines in the Cancer Cell Line Encyclopedia (https://www.cancer.gov/ccg/research/structural-genomics/tcga/history/policies/ccle-open-release-justification.pdf). For the Jurkat cell line, short-read whole- genome sequencing data were previously published^36^ without controlled access in public databases, so there is minimal additional risk of reidentification for publishing the long-read whole genome sequencing data in this manuscript. To make progress toward similar cell lines with explicit consent for public genome data sharing and cell line development, NIST is currently working toward establishing T cell lines and other types of cell lines from individuals who have provided consent under the Personal Genome Project.^37^ This consent includes permission to share genomic data and cell lines publicly for open research and commercial use, understanding the risks associated with this. For these reasons, the NIST Research Protections Office determined that the whole genome sequencing data generated in this work could be made publicly available, though it can not be shared for these cell lines due to restrictions in the MTA between NIST and Lentigen.

### Cell lines and cell culture

Parental Jurkat cell line (ATCC, Manassas, VA) and four clonal VCN Jurkat cell lines^29^ (kindly provided by Lentigen, Gaithersburg, MD) were cultured in RPMI-1640 growth medium (ATCC, Manassas, VA) supplemented with 10% heat-inactivated fetal bovine serum (FBS, Thermo Fisher Scientific, Grand Island, NY) and 2 mM Glutamax (Thermo Fisher Scientific, Grand Island, NY). Cultures were maintained at a cell concentration between 3 × 10^5^ and 1 × 10^6^ viable cells/mL by the addition of fresh medium at 37°C with 5% CO_2_.

### G-banded karyotyping

Karyotyping was performed by KaryoLogic Inc (Durham, NC) for all VCN clonal cells at passage 10. Approximately 1 million viable cells of each line were sent for karyotyping. Cytogenetic analysis was performed on 20 G-banded metaphase spreads for each clonal cell line.

### Genomic DNA extraction and purification

Large batches of cells were prepared from each cell line and used to prepare the genomic DNA. Briefly, the cells were collected by centrifugation at 300 × *g* for 5 min, and then washed twice with Dulbecco’s phosphate-buffered saline (DPBS). Large-scale DNA extraction and purification were accomplished using the modified Zymo Quick-gDNA midiPrep kit (Zymo Research Cat# D3100) protocol. All purified genomic DNA samples were eluted in TE buffer (10 mmol/L Tris, 1 mmol/L EDTA, pH 8.0) and stored at 4°C.

### Genomic DNA concentration and VCN candidate reference material packaging

DNA concentration and purity were determined by absorption measurements at 260 nm and 280 nm as in the previous preparation of NIST SRM 2373 and RM 8366. The VCN test material was prepared at 85 μL (approximately 25 ng/μL DNA) in 0.5 mL Sarstedt polypropylene tubes (Newton, NC, #72.730.105) for each of the five components.

### Fixed cell and cell plug candidate RMs

The cells were fixed with Carnoy’s solution (3:1 volume of methanol and acetic acid) according to MissionBio Tapestri Platform V2 – Preparation of Methanol fix cells for cell storage and Tapestri runs protocol (supplemental protocol I) or with formaldehyde according to Cergentis’ Sample Cross-link and Shipment v2 protocol. To prepare cell plugs, cells were collected and treated with Proteinase K (1 g/L) overnight, and then encapsulated in plugs of low melting temperature agarose according to the Genomic Vision’s instructions.

### Homogeneity assessment and stability analysis

The components of VCN candidate RMs were aliquoted into Sarstedt tubes (160 vials for each component) and were then stored at 4°C (range 4°C–6°C) in the dark. Homogeneity studies were performed by randomly selecting eight vials using the “R” program (set #2, 9, 33, 55, 67, 78, 88, and 102) of each component. The set numbers are the sequential numbers of the vials aliquoted during the vialing process. These samples were analyzed using the duplexed ddPCR assays for the reference genes (*RPL32* and S2) and the target genes (LTR and RRE).^29^ The VCN was calculated by 2-fold of the ratio of target sequence RRE in integrated provirus vector and reference gene *RPL32* in human genome.^29^ The stability of the DNA samples was evaluated by VCN measurements of randomly selected vials at each time point using the ddPCR assays up to 810 days.

### Interlaboratory study

Participants were recruited according to their ability to perform quantitative analysis of LV integration. A total of 12 participants were recruited for the interlaboratory study through the NIST genome editing consortium, an open announcement on the NIST website, and personal contacts. Three principal methods, i.e., qPCR, dPCR, and NGS were used by 11 laboratories for the quantification of LV integration copy numbers (see Tables 1 and 2 for instrumentation and method). One imaging-based method was also used to evaluate the LV integration site. Sets of blinded samples of DNA, fixed cells, or both were sent to participants as requested.

### Single-cell vector integration site assay

Single-cell vector integration site analysis was conducted according to a modified Mission Bio Tapestri single-cell DNA protocol. In summary, the Tn5 tagmentation methodology was employed to generate genome fragments in-drop with universal adaptors (Mission Bio “Seq8” adaptors). Using an LTR primer (with Mission Bio “Read2” adaptors) extending from the vector toward the genome, amplicons containing vector and human genome junctions (vector integration sites) were created and subsequently processed for NGS readout.

Tn5 adaptor: GCGAGCAGTGTCAGCAGATGTGTATAAGAGACAG.

Tn5 primer: GAGACATATCGACTAGCTGCGTATGCGAGCAGTTGCAGC.

LTR primer: ACTCGCAGTAGTCGATCCCTCAGACCCTTTTAGTC.

### Molecular combing

Molecular combing, an imaged-based method with a detection strategy called Genomic Morse Code (GMC), was used to directly visualize LV insertion in the genome.

#### GMC design

LV integration bins (insertion >3 kb) were analyzed using molecular combing technology. The GMC online software was employed to generate *in silico* probes spanning the anticipated integration regions, along with flanking probes located 10 kb upstream and 15 kb downstream of each site of interest. GMC design, a combination of probes of diverse colors (blue, red, and green in this study) specific to a genomic region, is interpreted as a pattern of dots and lines on the coverslip. GMC probes generation and design verification relies on BLAST (version ≥2.12.0) to mitigate potential issues related to cross-design and inner-design or off-target hybridizations and to optimize GMC enabling multiplexing on the same coverslip.

#### Molecular combing and staining

DNA fibers were obtained using the FiberPrep Kit (Genomic Vision). Extraction of DNA fibers was performed after agarose melting and overnight digestion by beta-agarase. DNA released from the plugs was stretched on treated coverslips at a constant rate using the Molecular Combing System (Genomic Vision). Hybridization was carried out overnight in a Hybridizer (Dako) with the labeled probes prior to detection using fluorophore-coupled antibody layers (Alexa Fluor 647-conjugated immunoglobulin [Ig]G anti-Digoxin and Cy3-conjugated IgG anti-fluorescein from Jackson ImmunoResearch; BV480 Streptavidin from BD Horizon). Coverslips were washed with 2X SSC with 1% Tween 20 and dehydrated with successive ethanol baths (70%, 90%, and 100%) before being placed on a sample holder (Genomic Vision). The entire coverslip was scanned on a FiberVision automated scanner (Genomic Vision), and image analysis and signals measurement were performed using FiberStudio software (Genomic Vision).

#### GMC detection and report algorithm

The GMC signals were detected using the pattern recognition module of FiberStudio, relying on their segmentation and classification. The Deep Learning model of semantic segmentation was trained on Genomic Vision’s image data from various assays. LinkNet convolutional neural network architecture with ResNet34 backbone was used for the model, labeling each pixel in the image with a corresponding probe color with a novel loss function adapted for generic GMC detection model training (Genomic Vision’s intellectual property). These classified signals were then reviewed and validated by operators. A data report with descriptive statistics of the GMC designs, patterns, and the probe lengths validated was generated for each coverslip and used for quantification and statistical analysis. Models and algorithms were developed by using TensorFlow (version ≥ v2.4) and Pytorch (version ≥ v1.7) libraries, under first python 3.8 and later python 3.10.

#### LV integration identification and statistical analysis

The length distribution of the probe within a population of signals from fully stretched fibers approximates a Gaussian distribution (Shapiro-Wilk test, Kolmogorov-Smirnov test, SciPy 1.7.3). Given this information, when dealing with samples exhibiting a substantial proportion of the LV integration (>5% in the mix with wild-type), signals corresponding to the wild-type GMC and LV integration GMC were separated using Gaussian Mixed Models (GMM) clustering applied to the probe lengths with default parameters (scikit-learn 0.24.2). When the LV integration is present at a lower proportion (<2%), all signals are considered to endorse the alternative GMC design when the signal length of the probe stray by 3 standard deviations (sigma) from the mean of the wild-type probe length, as approximated by the Gaussian distribution. The frequency of the searched LV integration is then quantified as the ratio of signals supporting the alternative GMC to the total number of signals.

### Whole genome sequencing

Whole genome sequencing analysis was used to detect lentiviral vector integration sites in the Jurkat cell line genome. The NGS libraries were prepared by using NEXTflex Rapid Library Prep Kit, and sequencing was performed on the Illumina HiSeq 4000 platform. Reads spanning an integration site in which one end of the sequence stemming from the integrated vector and the other anchored in the human genome, resulting in a partial alignment, were obtained using bowtie in local alignment mode. Two independent whole genome sequencing assays on sample A (VCN4) were performed to confirm the integration sites reported by other participating laboratories.

### PacBio long-read sequencing

The sizing of the isolated gDNA was checked and confirmed prior to library preparation. Libraries were prepared following the PacBio procedure and checklist by using SMRTbell prep kit 3.0" (PN: 102-166-600 REV02 MAR2023). Sequencing-ready SMRTbell libraries were sequenced on a Revio system using the following reagents and consumables: Revio sequencing plate (PN 102-587-400) and Revio SMRTcell tray (PN 102-202-200). The results were used to further confirm and validate the integration sites in sample A (VCN4).

#### PacBio read alignment

PacBio HiFi whole genome long-read sequencing was performed and used to identify LVV integration sites (ISs) in the Jurkat genome. Reads were mapped to a modified GIABv3 human GRCh38 human reference genome (https://ftp-trace.ncbi.nlm.nih.gov/ReferenceSamples/giab/release/references/GRCh38/GRCh38_GIABv3_no_alt_analysis_set_maskedGRC_decoys_MAP2K3_KMT2C_KCNJ18.fasta.gz), with the expected 4,154-bp provirus sequence added as an additional GRCh38 chromosome for mapping. The PacBio HiFi reads were mapped to the modified reference using pbmm2 (v1.13.1, https://github.com/PacificBiosciences/pbmm2), a minimap2 wrapper for PacBio reads, with default parameters.

#### PacBio variant calling

Variant calling was performed to identify integration sites and confirm the complete insertion of the provirus sequence. Insertions were identified using the PacBio structural variant caller, pbsv (v2.9.0, https://github.com/PacificBiosciences/pbsv). Variant calling, using default parameters, was performed using pbmm2 long-read alignments against the modified reference.

#### Identification of integration sites with manual curation

The Integrated Genome Viewer (IGV v.2.16.2) was used to identify and visualize integration sites using the modified reference and the VCN4 pbmm2 long-read alignments and pbsv structural variant calls. Some reads were aligned across the integration site and contained an insertion that matched the provirus sequence. Other reads were clipped at the integration site, and the remaining part of the read aligned to the provirus sequence. All reads mapping to the provirus chromosome had evidence of a single base insertion, “C” at position 830 and an SNP, A→G, at position 1363, demonstrating that the inserted sequences slightly differed from the expected provirus sequence. To identify putative integration sites, we looked at the coordinates of the supplementary alignments associated with reads aligning to the provirus chromosome. Four genomic regions were noted as possible integration sites. These four regions were curated, and four insertion variant calls were observed with supporting read alignments. All pbsv insertion calls identified a 4,160-bp insertion. Further, the integration sites showed an increase in coverage over 5 bp, consistent with duplication of the lentiviral integration site sequence (as shown in Table 6). Further confirmation of integration was performed with the Genome Ribbon (https://github.com/MariaNattestad/Ribbon) complex variant visualization tool. VCN4 alignments and pbsv variants were loaded and visualization of the provirus chromosome alignments showed reads aligning across the provirus chromosome and the four previously identified GRCh38 genomic regions.

**Table 6:** Identification of VCN4 integration sites with manual curation

GRCh38 genomic coordinates	5-bp integration site sequence	pbsv ID	Provirus insertion length (bp)
chr22: 42,082,672 - 42,082,676	GTTTT	pbsv.INS.54478	4160
chr16: 2,259,698 - 2,259,702	GCTAA	pbsv.INS.43848	4160
chr1:160,440,776 - 160,440,780	GTACA	pbsv.INS.2597	4160
chr1: 45,634,495 - 45,634,499	GTTTG	pbsv.INS.110	4160

#### Provirus insertion confirmation

To confirm full insertion of the provirus sequence and that no partial insertions were missed, all pbsv-identified insertions greater than 50 bp were aligned back to the modified reference. These alignments were visualized in IGV and only the four previously identified insertions aligned to the provirus chromosome. Moreover, all insertions were found to be full length, 4,155 bp, which includes the 1-bp insertion.

#### VCN4 integration site analysis summary

We performed PacBio HiFi long-read whole genome sequencing on VCN4 to further confirm the insertion sites and insertion sequences. We confirmed the four insertion sites, did not find additional full or partial insertions, and confirmed the full 4,154 bp were inserted as expected, except all insertions had one extra base inserted at position 830 in the insert.

### Statistics

The NIST Consensus Builder (https://consensus.nist.gov/app/nicob, accessible on Nov 27, 2024) was used to estimate the consensus value of NIST candidate RMs from dPCR assays. The DerSimonian-Laird procedure^38^ was employed to calculate consensus values and the set-to-set variability in the interlaboratory portion of this study. It was also used to assess the set-to-set variability in the homogeneity analysis of this material.

The DerSimonian-Laird approach combined estimates from each lab (or set, in the homogeneity assessment) while giving more weight in the pooled estimate to results with lower uncertainties, ensuring more robust results. It produced a standard uncertainty estimate (i.e., the standard error for the reported consensus value) as well as a "dark uncertainty" estimate. In the analyses of interlaboratory data, dark uncertainty provided an estimate of the standard deviation for random lab effects based on the extent to which the observed variability between individual estimated values exceeds what would be expected given each value’s respective uncertainty assessment. During the homogeneity assessment, dark uncertainty estimated the standard deviation for random set effects. In either application, a dark uncertainty estimate of 0 would indicate that data show no signs of relative biases between laboratories (or sets), which would correspond to the ideal case where random measurement noise was the only source of variability.

## Data availability

The data supporting the findings of this study are available from the corresponding authors (H.-J.H., Z.H., or S.L.-G.) upon reasonable request. The NGS data are not publicly available due to restrictions in the material transfer agreement between NIST and Lentigen.

## Acknowledgments

In-kind donations of clonal VCN Jurkat cell lines to support this specific study were provided to the 10.13039/100000161National Institute of Standards and Technology by Lentigen. A.S.W., D.Y., and C.A.F. acknowledge financial support from the UK Department for Science, Innovation and Technology (10.13039/100031278DSIT) through the Chemical and Biological Metrology (CBM) program. Certain commercial equipment, instruments, or materials (or suppliers or software) are identified in this paper to foster understanding. Such identification does not imply recommendation or endorsement by the National Institute of Standards and Technology, nor does it imply that the materials or equipment identified are necessarily the best available for the purpose.

## Author contributions

NIST developed candidate RMs and coordinated the interlaboratory study. H.-J.H., Z.H., and S.L.-G. led the interlaboratory study. In addition, H.-J.H. and Z.H. carried out the experiments and analyzed the data. S.P.L. provided statistical analysis. J.H.M. and J.M.Z. analyzed NGS data. S. Miller performed data management. S. Maragh coordinated this study. A.T. performed cell culture and prepared DNA samples. M.M. and S.P. prepared fixed cell samples. K.D.C. and S.L.-G. initiated communications with Lentigen for the clonal VCN cell line transfers. B.D. and B.S.P. from Lentigen developed clonal VCN cell lines. L.T., Y.Q., and D.C.C. generated Azenta NGS data and performed analysis. B.D., R.M., and O.S. generated CaringCross qPCR data and performed analysis. P.W. generated Castle Creek Bio qPCR data and performed analysis. E.S., G.D., and H.F. generated Cergentis NGS and dPCR data, and performed analysis. S.A.-S., R.M., and E.A. generated Genomic Vision molecular combing data and performed analysis. D.S., J.S., and M.R. generated Glycostem qPCR data and performed analysis. S.S. generated GSK dPCR data and performed analysis. C.-Y.L., and B.S. generated MissionBio NGS and dPCR data, and performed analysis. A.S.W. and D.Y. generated NML at LGC dPCR data and performed analysis. C.A.F. performed analysis. S.N., F.T., M.S., and Y.D. generated Sampled dPCR data and performed analysis. J.K. and U.L. generated Thermo Fisher Sci dPCR data and performed analysis. H.-J.H., Z.H., and S.L.-G. wrote the manuscript with input from all the authors.

## Declaration of interests

The authors declare no competing interests.

## Supplemental information

Document S1. Figures S1–S3, Tables S1–S3, and Supplemental Protocol

Table S2. Karyotyping results of clonal VCN cell lines Cytogenetic analysis was performed on 20 G-banded metaphase spreads for each clonal cell line.

Document S2. Article plus supplemental information
