# Supplementary material for: Interlaboratory assessment of candidate reference materials for lentiviral vector copy number and integration site measurements
Source: Mol Ther Methods Clin Dev. 2025 Apr 21;33(2):101472. doi: 10.1016/j.omtm.2025.101472 (PMC12229725; doi:10.1016/j.omtm.2025.101472)
Supplement: Document S1. Figures S1–S3, Tables S1–S3, and Supplemental Protocol [file mmc1.pdf]

OMTM, Volume *33*

Supplemental information

Interlaboratory assessment of candidate reference materials for lentiviral vector copy number and integration site measurements

Hua-Jun He, Zhiyong He, Steven P. Lund, Laure Turner, Yongjun Fan, Yu Qiu, David C. Corney, Boro Dropulic, Rimas Orentas, Oxana Slessareva, Priscilla Welch, Katie Dungca, Ellen Stelloo, Gabrielle Dijksteel, Harma Feitsma, Sana Ahmed-Seghir, Rostyslav Makarenko, Engin Altunlu, Daniëlle Steenmans, Jan Spanholtz, Monica Raimo, Shai Senderovich, Barbara S. Paugh, Chieh-Yuan Li, Benjamin Schroeder, Alexandra S. Whale, Dilek Yener, Carole A. Foy, Shareef Nahas, Feng Tu, Michael Sheldon, Yan Ding, Jennifer Kandell, Uma Lakshmipathy, Jennifer H. McDaniel, Justin M. Zook, Sierra Miller, Samantha Maragh, Simona Patange, Mahir Mohiuddin, Alessandro Tona, Kenneth D. Cole, and Sheng Lin-Gibson

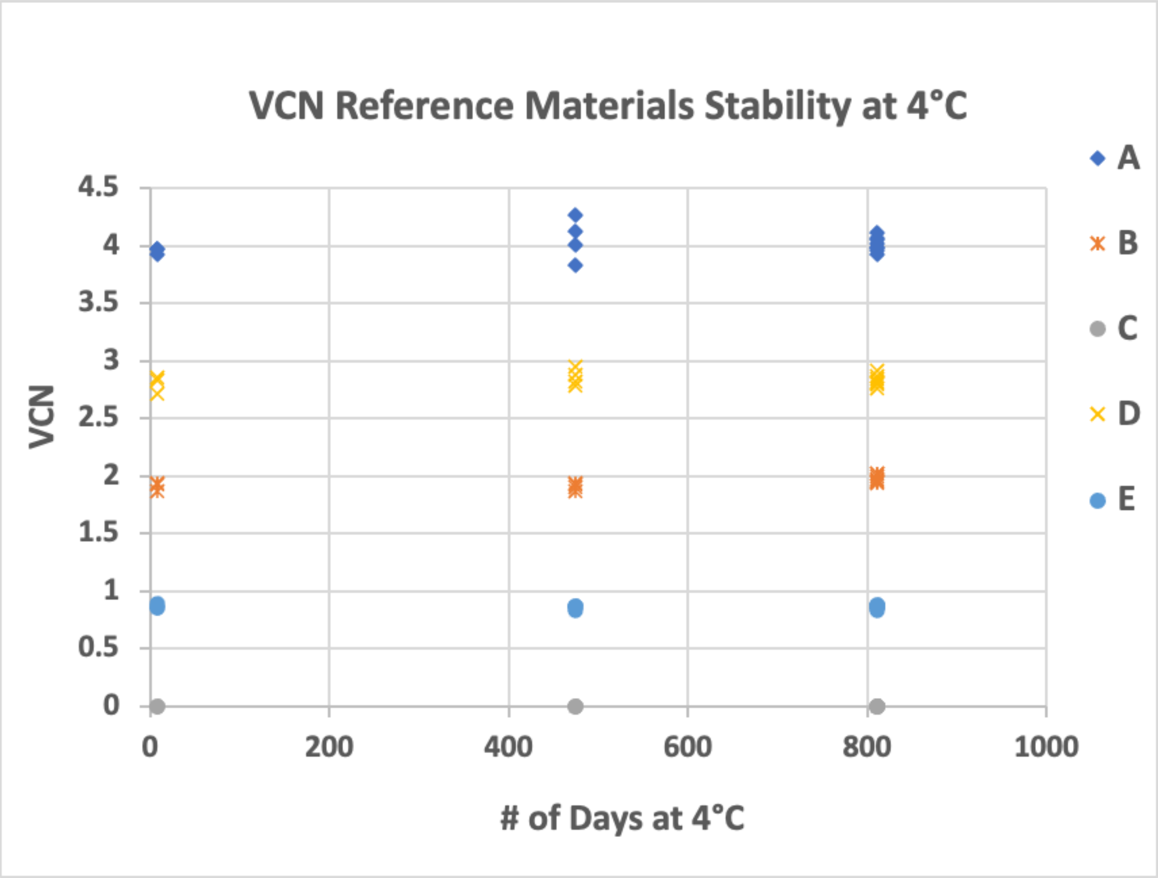

Figure S1. The stability of VCN candidate reference materials
VCN was measured by Bio-Rad droplet dPCR followed for different periods of time on samples stored at 4°C (range 4-6°C) in the dark. Individual sample was measured in four replicates.

A.

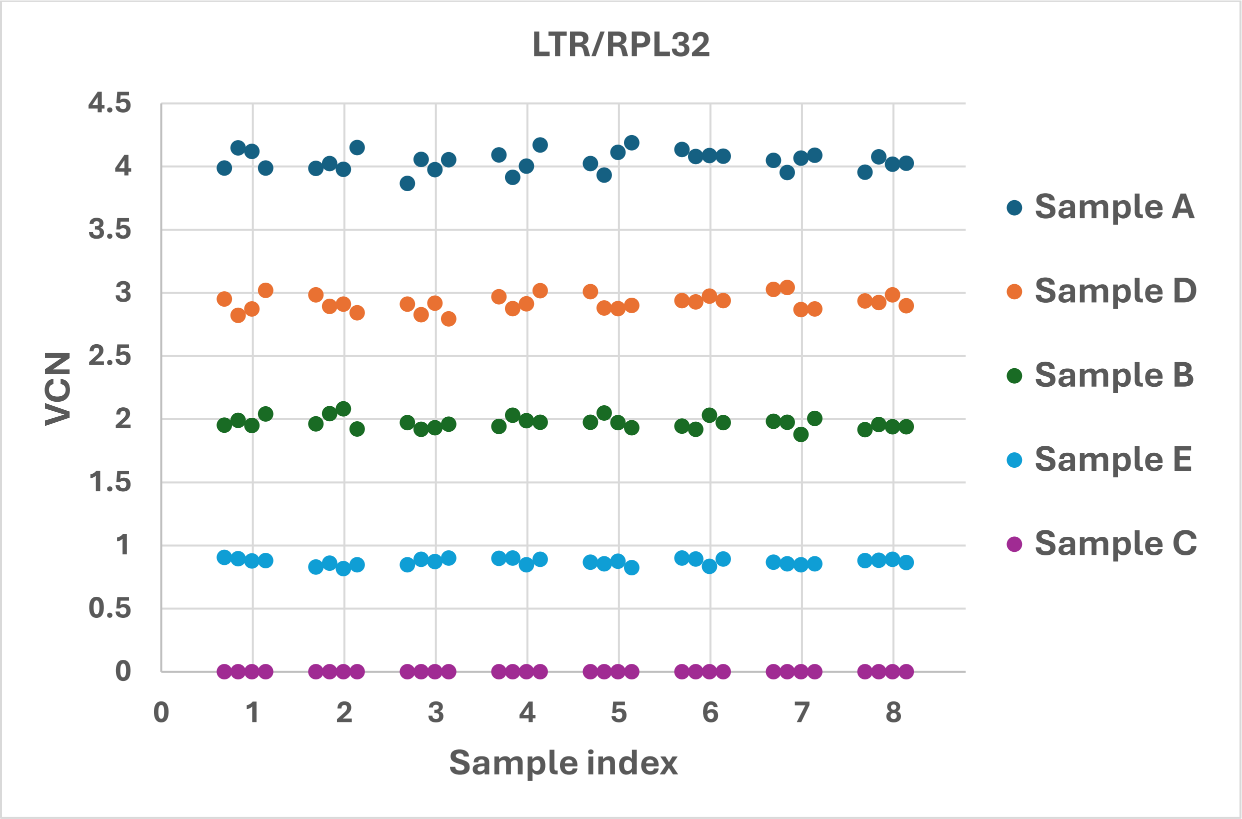

LTR/RPL32
VCN
4.5
4
3.5
3
2.5
2
1.5
1
0.5
0
0
1
2
3
4
5
6
7
8
Sample index
Sample A
Sample D
Sample B
Sample E
Sample C

B.

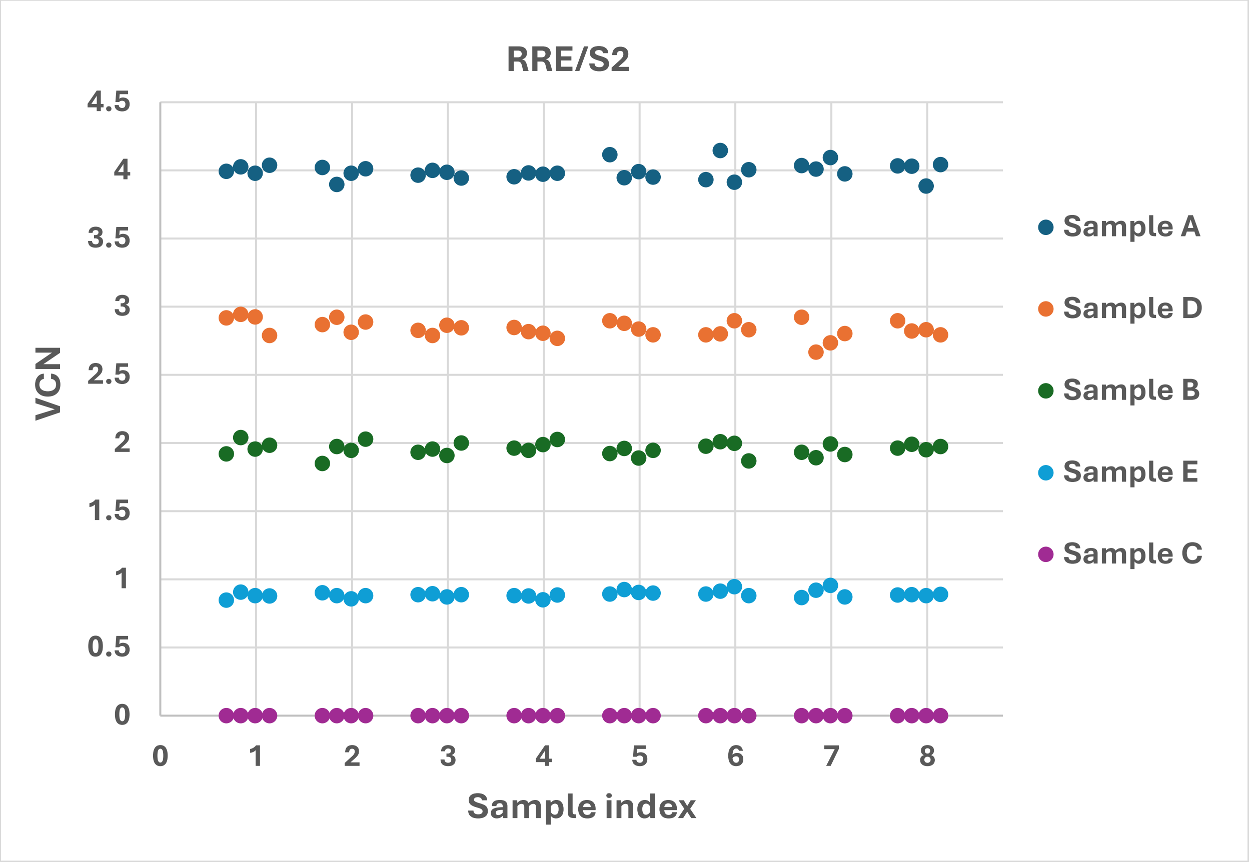

Figure S2. The homogeneity study of VCN candidate reference materials
Randomly selected sample set: 1) Set 2. 2) Set 9. 3) Set 33. 4) Set 55. 5) Set 67. 6) Set 78. 7) Set 88. 8) Set 102. A). Assay of LTR/RPL32; B). Assay of RRE/S2.

VCN per cell	Clone Number	Provirus integration site
0	J0-1	N/A, parental cell line
1	J5-3	chr3: 35632784
2	J22-1	chr6: 45210754
		chr22: 40844112
3	J18-3	chr3:130653830
		chr14:23033347
		chr15:25370116
4	J8-2	chr1:46100171
		chr1:160410538
		chr4:5614465
		chr22:42478680

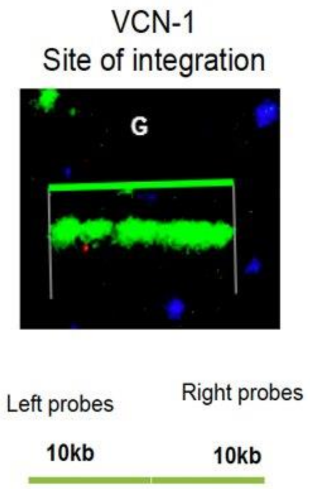

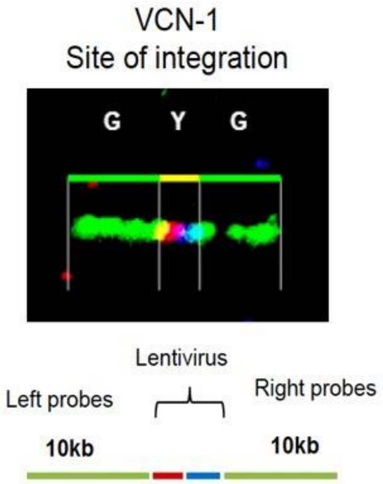

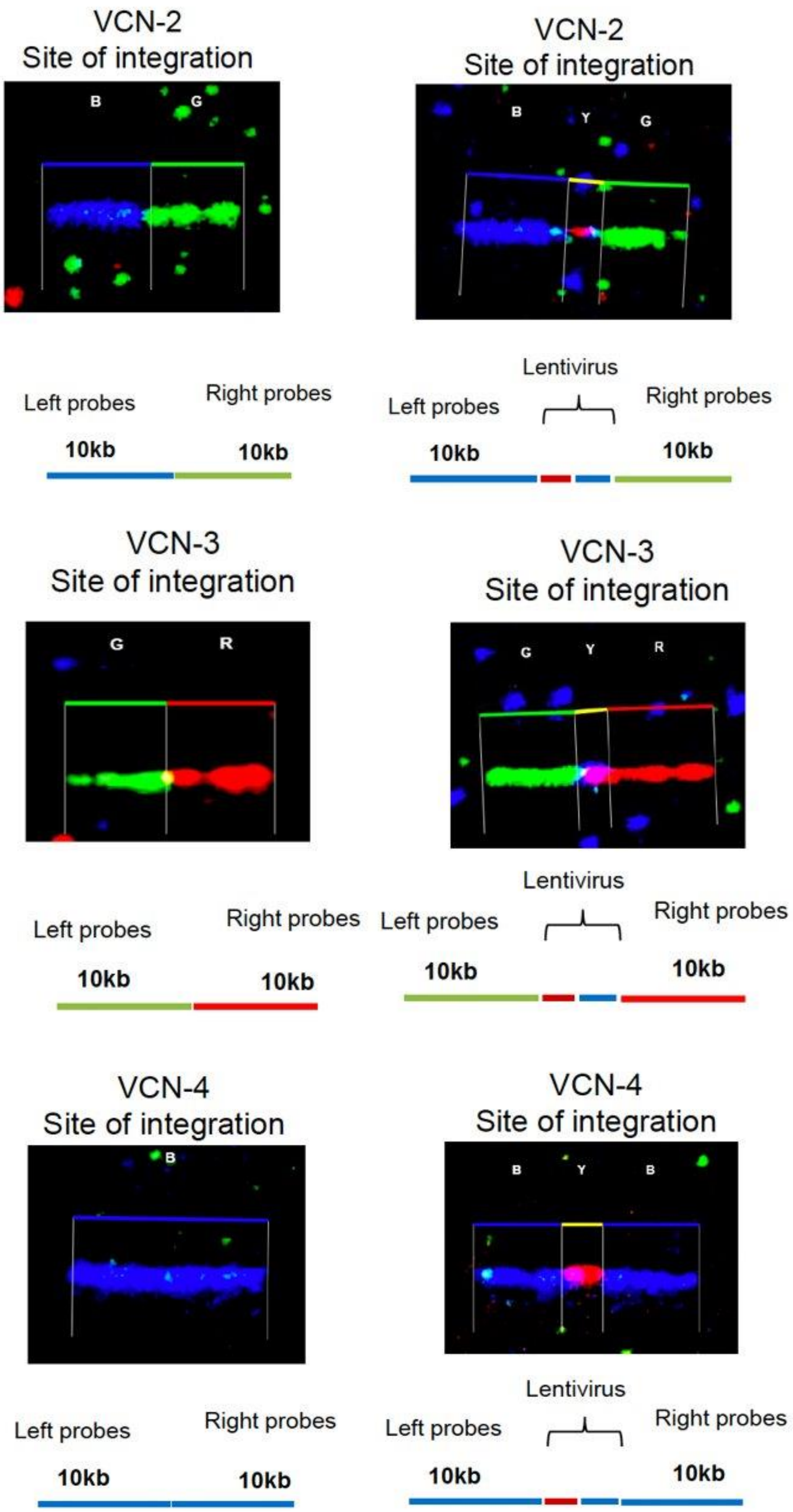
VCN-2
Site of integration
B
G
Left probes
Right probes
10kb
10kb
VCN-2
Site of integration
B
Y
G
Lentivirus
Left probes
Right probes
10kb
10kb
VCN-3
Site of integration
G
R
Left probes
Right probes
10kb
10kb
VCN-3
Site of integration
G
Y
R
Lentivirus
Left probes
Right probes
10kb
10kb
VCN-4
Site of integration
B
Left probes
Right probes
10kb
10kb
VCN-4
Site of integration
B
Y
B
Lentivirus
Left probes
Right probes
10kb
10kb

Figure S3. Integration Sites Determined by Direct Visualization (Lab 12)
Cell plugs were prepared for molecular combing technology. One integration site was chosen for each clonal VCN cell line (highlighted in red). Fluorescence labeled probes were used to direct visualize the integration sites.

Table S1. Homogeneity assessment by Dersimonian-Laird analysis.
The standard deviation corresponding to random set effects (i.e., set-to-set variability) was analyzed by Dersimonian Laird analyses. It produced a standard uncertainty estimate (i.e., the standard error for the reported consensus value) as well as a "dark uncertainty" estimate.

Sample	Target	Estimate	tau	Set_cv (%)
**A**	LTR/ RPL32	4.05	0.03	0.74
**A**	RRE/ S2	3.99	0.01	0.31
**B**	LTR/ RPL32	1.96	0.02	0.77
**B**	RRE/ S2	1.96	0.01	0.46
**C**	LTR/ RPL32	0.00	0.00	0.00
**C**	RRE/ S2	0.00	0.00	0.00
**D**	LTR/ RPL32	2.93	0.01	0.38
**D**	RRE/ S2	2.84	0.01	0.45
**E**	LTR/ RPL32	0.87	0.02	1.92
**E**	RRE/ S2	0.89	0.01	0.70

Table S2. Karyotyping results of clonal VCN cell lines.
Cytogenetic analysis was performed on twenty G-banded metaphase spreads for each clonal cell lines.

Table S3. NGS based technologies and methods used by interlaboratory study participants.

Candidate RMs tested by participants with NGS assays are reported in this table.

Lab	Sequencing platform	Enrichment method (if any)	Sequencing strategy	Library construction protocol	Material Requested
**9**	Illumina Nextseq platform (NextSeq flowcells (v2.5))	Targeted Locus Amplification (TLA)	Targeted sequencing	Nextera XT reaction	Fixed cells
**10**	Illumina Nextseq	Tapestri	Targeted sequencing	Mission Bio DNA V2 protocol	Fixed cells
**11**	Illumina Miseq	Capture based NGS (LTR primer)	Targeted sequencing	Using index nested LTR specific primer and index sample specific primer	DNA

Supplemental Protocol

Preparation of Methanol fix cells for cell storage and Tapestri runs protocol.

Carnoy's Fixative preparation
Reagent Volume
Methanol 90 mL
Acetic Acid 30 mL
Total 120 mL

Prepare Carnoy's Fixative in the fume hood with proper PPE (nitrile gloves, lab coat, safety goggles). Place the glass of the fume hood between yourself and the solution, or alternatively wear a face shield. Once prepared, store the Carnoy's fixative on ice or 4C.

Fixation of suspension cells (Jurkat)

1. Count cells and transfer a volume equivalent to 60 mil (to account for loss during centrifugation steps) cells to a 15 ml tube.
2. Centrifuge cells at 200 g for 5 min at RT.
3. Discard supernatant in biohazard waste.
4. Add 10 ml of DPBS+0.1% BSA and resuspend pellet.
5. Strain suspension through a 40 µm strainer into a new 50- ml tube (the strainer fits easily into the tube).
6. Centrifuge cells at 200 g for 5 min.
7. Remove supernatant and discard in biohazard waste.
8. Add 5 ml of DPBS and resuspend the cell pellet by gentle pipetting.
9. Divide between 2 50-ml tubes (2.5 ml/each) and transfer cell suspensions to the fume hood.
10. Prepare fresh Carnoy's Fixative (follow recipe) in the fume hood while wearing proper PPE (see notes below).
11. Place a vortex in the fume hood and within secondary containment. Set the vortex on Auto, speed at 2.5, and pipettor dispensing button on Slow
12. Place cell suspension on vortex and start vortexing.
13. **Drop by drop, add 25ml of Carnoy's fixative to the cells while vortexing**. Tighten the cap on the tubes to avoid spillage and keep cells on ice after fixative is added.

14. Centrifuge the cells at **400** g for 10 min. I**<u>MPORTANT</u>**: when spinning down in 50-ml tube, increase g or the cells will not form a nice pellet. If after centrifugation you can't see a nice pellet or if in doubt, repeat centrifugation step.

15. Bring the tubes back into the fume hood and remove supernatant as organic waste (see notes below).

16. In the fume hood, add enough Carnoy's Fixative to get the desired cell concentration e.g. 25 mL of fixative to get $1x10^6$ cells/mL.

17. Pool the contents of the 2 tubes into 1

18. Optionally, aliquot 1mL of cell suspension in fixative into 1.5mL Eppendorf tubes. Make sure caps are secure before removing the samples from the fume hood.

19. Label the tubes and store the cells at 4C for up to 1 year.
